# Intracardiac echocardiography versus fluoroscopy for endovascular and endocardial catheter navigation during cryo-ablation of the slow pathway in AVNRT patients

**DOI:** 10.1186/s12947-019-0162-2

**Published:** 2019-06-11

**Authors:** Blerim Luani, Thomas Rauwolf, Conrad Genz, Alexander Schmeißer, Marcus Wiemer, Rüdiger C. Braun-Dullaeus

**Affiliations:** 1 Department of Cardiology and Intensive Care Medicine, Johannes Wesling University Hospital, Ruhr University Bochum, Hans-Nolte-Str. 1, 32429 Minden, Germany; 2 Department of Internal Medicine, Division of Cardiology and Angiology, Magdeburg University, Leipzigerstr. 44, 39120 Magdeburg, Germany

**Keywords:** Intracardiac echocardiography, Zero-fluoroscopy, AVNRT, Cryo-ablation, Catheter ablation

## Abstract

**Background:**

A new zero-fluoroscopy technique for electrophysiology catheter navigation relying on intracardiac echocardiography (ICE) has been recently reported (Ice&ICE trial). We investigated potential differences in efficacy, safety or procedural performance between conventional fluoroscopy- and ICE-guided cryothermal ablation (CA) in symptomatic AVNRT patients.

**Methods:**

Clinical and electrophysiological data of AVNRT patients included in the Ice&ICE trial (22 patients, 16 females; =zero-fluoroscopy group) were compared to those of consecutive AVNRT patients, who underwent fluoroscopy-guided CA (25 patients, 17 females; = fluoroscopy group) during the last 2 years in our institution.

**Results:**

Slow pathway ablation or modulation was successful in all patients. Fluoroscopy time and radiation dose in the fluoroscopy group were 11.2 ± 9.0 min and 20.3 ± 16.2Gycm^2^, whereas no fluoroscopy was used in the opposite group (*p* <  0.001, respectively). EPS duration was not different between the groups (zero-fluoroscopy:101.6 ± 40.2 min, fluoroscopy:99.4 ± 37.2 min, *p* = n.s.). Catheter placement time was significantly shorter in the fluoroscopy group (2.2 ± 1.6 min vs. 12.0 ± 7.5 min, *p* <  0.05), whereas cryo-application duration (from the first cryo-mapping to the last CA) was significantly shorter in the zero-fluoroscopy group (27.5 ± 37.0 min vs. 38.1 ± 33.9 min, *p* <  0.05). Mean cryo-mapping and CA applications were numerically lower in the zero-fluoroscopy group (CM:7.5 ± 5.7 vs. 8.8 ± 6.2; CA:3.1 ± 1.7 vs. 3.2 ± 2.0, *p* = n.s.). No major adverse events occurred in both groups. After 15.0 ± 4.2 months, arrhythmia recurrence was not different between the groups (4.5% vs. 8.0%, *p* = n.s.).

**Conclusions:**

Zero-fluoroscopy ICE-guided EP catheter navigation shows comparable efficacy and safety to fluoroscopic guidance during CA in AVNRT patients. ICE visualization of catheters and endocardial structures within the triangle of Koch shortens the cryo-application duration, though time needed for catheter placement is longer, when compared with conventional fluoroscopic guidance, which results in similar mean EPS duration with both navigation techniques.

**Trial registration:**

(German Clinical Trials Register ID: DRKS00011360; Registration Date 14.12.2016)

**Electronic supplementary material:**

The online version of this article (10.1186/s12947-019-0162-2) contains supplementary material, which is available to authorized users.

## Background

Catheter ablation of the slow pathway for treatment of symptomatic patients with atrioventricular-nodal-reentry-tachycardia (AVNRT) is highly effective and safe [1]. Usually, the endovascular and endocardial navigation of the diagnostic and ablation catheters is guided by fluoroscopy. Though fluoroscopy time and radiation dose have been substantially reduced by increasing operator experience and technical improvements, the long-term complications of ionizing radiation exposure during electrophysiological studies (EPS) to both patients and laboratory staff could not be neglected [2–6]. Therefore, alternative navigation techniques, which avoid or minimize fluoroscopy, are of great interest. With the event of three dimensional electroanatomical mapping (3D EAM) techniques, zero- or near-zero-fluoroscopy catheter ablation of most cardiac arrhythmias has been successfully performed in electrophysiology laboratories [7–10]. These navigation techniques enable the visualization of diagnostic and ablation catheters within reconstructed anatomical structures, facilitating the localization of the ablation substrate.

Recently, we reported the feasibility of a new zero-fluoropscopy approach for slow pathway ablation in symptomatic AVNRT patients using solely two- or three-dimensional intracardiac echocardiography (ICE) for catheter navigation [11]. Potential advantages of this technique over 3D EAM could be the real-time visualization of true endovascular borders (including duplex mediated blood flow direction), endocardial structures and diagnostic or ablation catheters, as well as immediate recognition of potential complications (such as pericardial effusion or thrombus formation). A drawback of the ICE guided navigation is the employment of an adjunctive catheter [8F for two-dimensional ICE guidance (AcuNav™, Biosense Webster, Inc) or 10F for three-dimensional ICE guidance (AcuNav™ V, Siemens AG, Munich, Germany)].

The broad implementation of new navigation techniques for AVNRT ablation should not be at the expense of a compromised safety or efficacy, when compared to the well-established conventional fluoroscopic catheter guidance. For this reason, we aimed to investigate in a retrospective comparison potential differences between the two catheter navigation techniques with respect to acute and long-term success rate, duration of EPS or different procedure steps and periprocedural complication rates.

## Methods

In our Ice&ICE Trial [11], which has investigated the feasibility of zero-fluoroscopy cryothermal ablation (CA) of the slow pathway guided by ICE navigation of the electrophysiology (EP) catheters, we included consecutive patients with documented and/or typical clinical manifestation of supraventricular tachycardia suggesting AVNRT after giving their written informed consent. The prospectively collected clinical and electrophysiological data of these patients were retrospectively compared to those of consecutive patients, who underwent fluoroscopy guided CA of the slow pathway during the last 2 years in our institution. The study was approved by the local ethics committee and was registered in the German Clinical Trial Register (DRKS00011360).

### ICE guided EPS

**Fig. 1:**
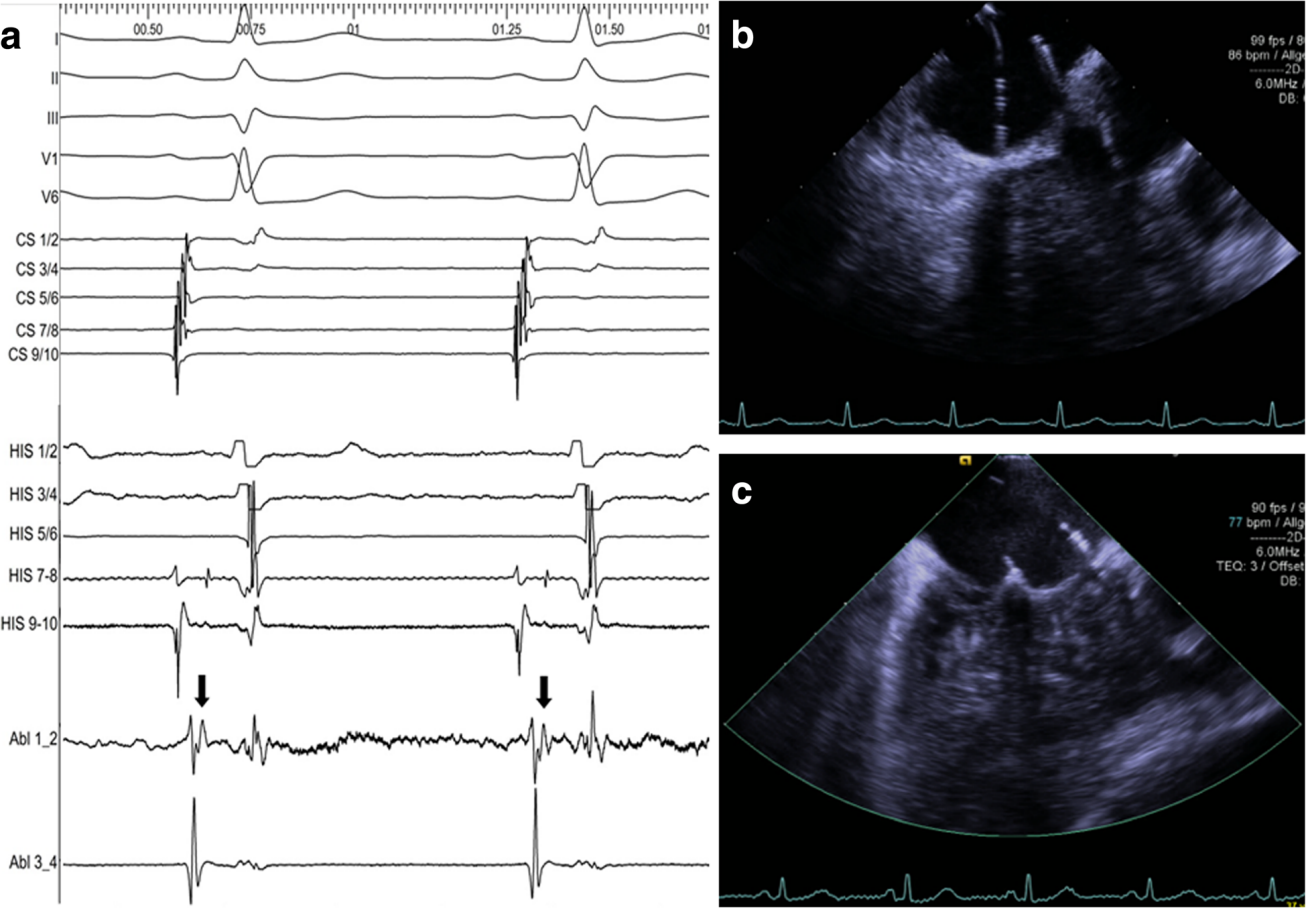
**a** Surface and endocardial ECG; CS panel showing atrial (1/2–9/10) and ventricular (1/2–7/8) potentials; His panel showing His (7/8, 9/10) and ventricular potentials (1/2–9/10); ablation catheter panel showing slow pathway potentials (black arrows); jagged isoelectric line (Abl 1/2) indicates ongoing cryo-mapping. **b** Cryo-mapping at the inferior triangle of Koch (ECG reveals uncompromised AV conduction) **c** Cryoadhesion during midseptal cryo-ablation - tenting of the septal tricuspid valve leaflet (entrapped by ice formation around the tip of the ablation catheter); distance to the His/RV diagnostic catheter on the right can be evaluated (ECG reveals uncompromised AV conduction)

We described the ICE guided navigation of the EP catheters previously in detail [11]. Shortly, a two- (8F) or three-dimensional (10F) ICE catheter was introduced through an appropriate sheath to visualize endovascular and endocardial borders and help the navigation of the EP catheters within these structures (see Additional file 1: Clip S1 and Additional file 2: Clip S2 for illustration). We implemented two 6F decapolar steerable catheters (Inquiry™; Abbott, Illinois, USA) to perform the diagnostic EPS, one placed in the coronary sinus and the second into the right ventricle across the anteroseptal aspect of the tricuspid ring (recording ventricle potentials with the distal and His potentials with the proximal electrodes, Fig. 1a. Cryo-mapping (CM; = freeze at − 30 °C, allowing reversible alteration of the ablation substrate and anticipation of the CA result, including evaluation of the AV- conduction integrity) and CA were performed with a 7F, 6-mm tip, steerable cryo-ablation catheter (Freezor Xtra®, 55 or 60 mm, Medtronic, Minneapolis, USA; Fig. 1b and c). Staff and patients did not primarily wear x-ray protection equipment during the ICE-guided EPS, but they were available if fluoroscopy had become necessary upon the discretion of the investigator. The duration of different procedural steps was prospectively assessed and defined as follows: sheath placement time (= time interval from the first vein puncture to the placement of the last sheath), EP catheter placement time (= time interval from the placement of the last sheath to the first stimulation manoeuvre), pre-ablation diagnostic evaluation time (= time interval from the first stimulation manoeuvre to the first CM), cryo-application time (= time interval from the beginning of the first CM to the end of the last CA), post-ablation diagnostic evaluation time (= time interval from the end of the last CA to removal of the catheters and sheaths, including the post-ablation waiting period).

### Fluoroscopy-guided EPS

Electrophysiological data of the patients in the fluoroscopy group were collected from the archived EP studies and protocols as well as from the cryo-application data archived on the CryoConsole™ (Medtronic, Minneapolis, USA) after adjusting the time difference between the EP system and CryoConsole™.

In the fluoroscopy group we implemented three diagnostic and one CA catheter (6F, decapolar stearable catheter in the coronary sinus; 5F, 4 polar catheter at the His position; 4F, 4 polar catheter in the RV apex; Freezor Xtra® for CM/CA, 55 or 60 mm). Sheath and EP catheter placement time, pre- and post-ablation diagnostic evaluation time and the cryo-application time were identically defined as in the ICE guided group.

After induction and confirmation of AVNRT as the clinical tachycardia, CA of the slow pathway was attempted in both groups targeting the slow pathway potentials, as previously described by Haissaguerre and Jackman [12, 13]. If no typical slow pathway potentials could be found, CM and CA were performed at typical slow pathway localization within the triangle of Koch (= anatomical approach). CA was always anticipated by a safe and effective CM (= uncompromised AV-conduction and abolition of slow pathway conduction or no AVNRT induction). After successful ablation (= abolition) or modification (= no repetitive conduction = max. one atrial echo) of the slow pathway, control stimulation was repeated after a waiting period of 20-30 min. Patients were monitored by ECG overnight before discharge and clinical follow up was collected from the scheduled outpatient visits after 3 and 12 months, according to our institutional routine practice after ablation therapy.

### Statistical analysis

Categorical parameters are presented as counts and percentages and were compared by Pearson’s chi-square test. Continuous variables are presented as mean values ± SD and were compared using the Kruskal-Wallis non-parametrical ANOVA. A *p*-value < 0.05 was considered statistically significant. Statistical analysis was performed using SPSS Version 22 (IBM, Armonk, NY, USA).

## Results

Twenty-seven consecutive patients were included in the ICE&Ice trial. AVNRT was inducible in 22 (81.5%; 16 female) patients (= zero-fluoroscopy group), whereas in the remaining five patients other or no supraventricular tachycardia could be induced (2 pts. with circus movement tachycardia, 1 pt. with focal atrial tachycardia and 2 pts. with no supraventricular tachycardia induction). During the last 2 years in our institution, fluoroscopy-guided CA of the slow pathway was performed in 25 (17 female) symptomatic AVNRT patients (= fluoroscopy group).

Patients clinical and electrophysiological parameters (shown in Table 1) were similar between the two groups, except for mean age, which was younger in the zero-fluoroscopy group (54.1 ± 11 years vs. 59.8 ± 12 years, *p* <  0.05).

**Table 1:** Patients clinical and electrophysiological parameters for both groups

	Zero-fluoroscopy group	Fluoroscopy group	*p* Value
Patients, *n*	22	25	
Mean age, years	54.1 ± 11	59.8 ± 12	< 0.05
Female, % (*n*)	72.7 (16)	68.0 (17)	n.s.
Fluoroscopy time in min.	0	11.2 ± 9.0	< 0.05
Radiation dose in Gycm^2^	0	20.3 ± 16.2	< 0.05
AVNRT type, % (n)			
slow/fast	86.4 (19)	88.0 (22)	n.s.
slow/slow	9.1 (2)	0	
fast/slow	4.5 (1)	3	
Orciprenaline needed for induction, % (n)	54.5 (12)	52.0 (13)	n.s.
AVNRT cycle length, ms	357 ± 55	349 ± 62	n.s.
PQ interval baseline, ms	153 ± 21	150 ± 19	n.s.
PQ interval post-abl., ms	152 ± 22	151 ± 20	n.s.
Antegrade Wenckebach baseline, ms	328 ± 36	324 ± 42	n.s.
Antegrade Wenckebach post-abl., ms	314 ± 38	326 ± 40	n.s.
DAVNP^a^ at baseline, % (n)	90.1 (20)	92.0 (23)	n.s.
DAVNP post-ablation, % (n)	45.5 (10)	44.0 (11)	n.s.

^a^*DAVNP* dual atrio-ventricular node physiology (= AH or VA jump of ≥50 ms)

Diagnostic EPS and CA of the slow pathway could be accomplished with no need for fluoroscopy in all AVNRT patients in the zero-fluoroscopy group, whereas fluoroscopy time and radiation dose in the fluoroscopy group were 11.2 ± 9.0 min and 20.3 ± 16.2 Gycm^2^, (*p* <  0.001, respectively).

Cryothermal ablation (= no conduction) or modulation (= no repetitive conduction = max. one atrial echo) of the slow pathway was primarily successful in all patients with inducible AVNRT in both groups (slow pathway ablation: *n* = 26, 55.3%; slow pathway modulation: *n* = 21, 44.7%). The proportion of patients with residual dual AV node physiology (DAVNP) but non-repetitive slow pathway conduction after the ablation procedure was not significantly different between the two groups, see Table 1 for details.

**Fig. 2:**
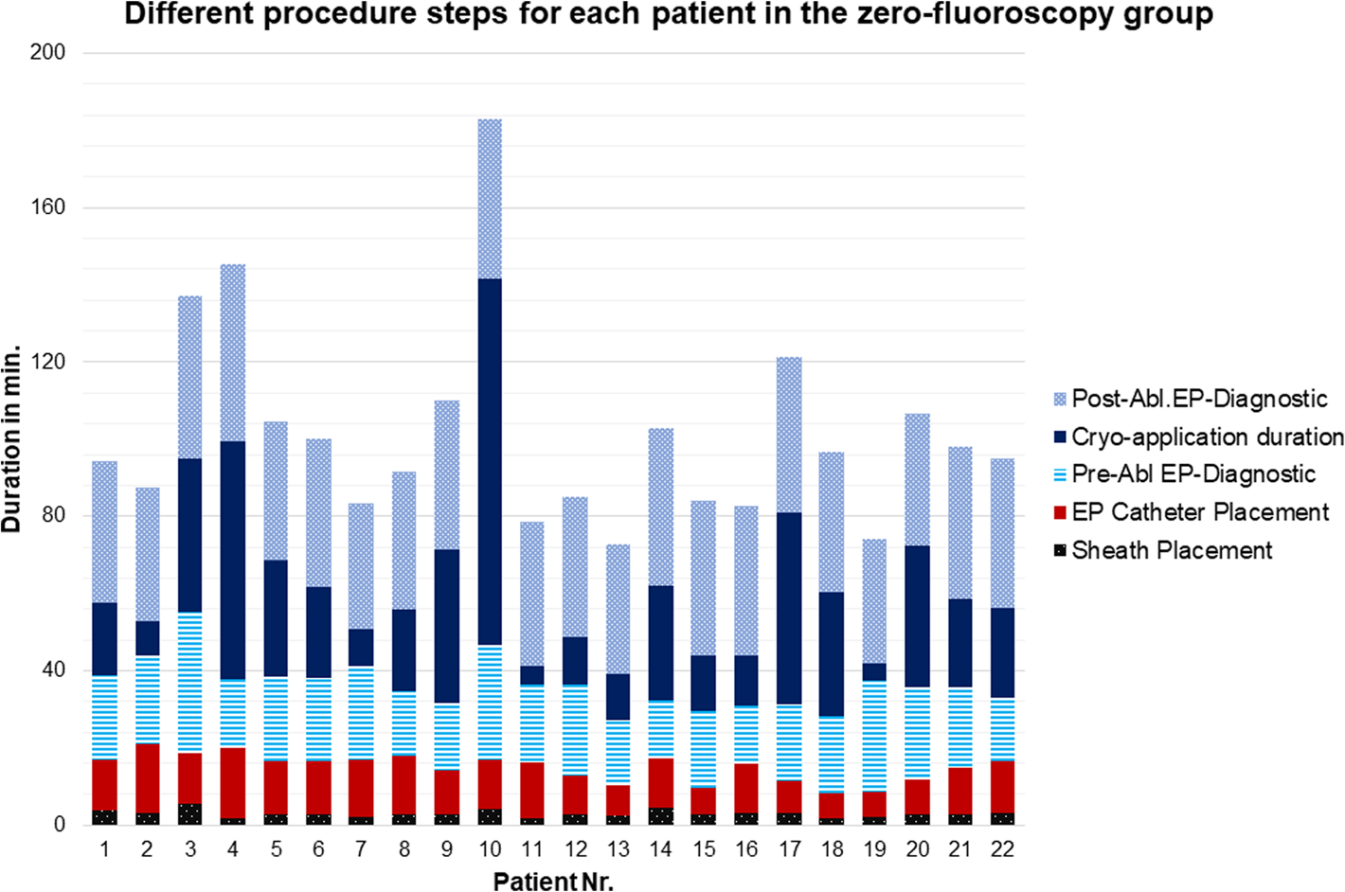
Different procedure steps for each patient in the zero-fluoroscopy group

**Fig. 3:**
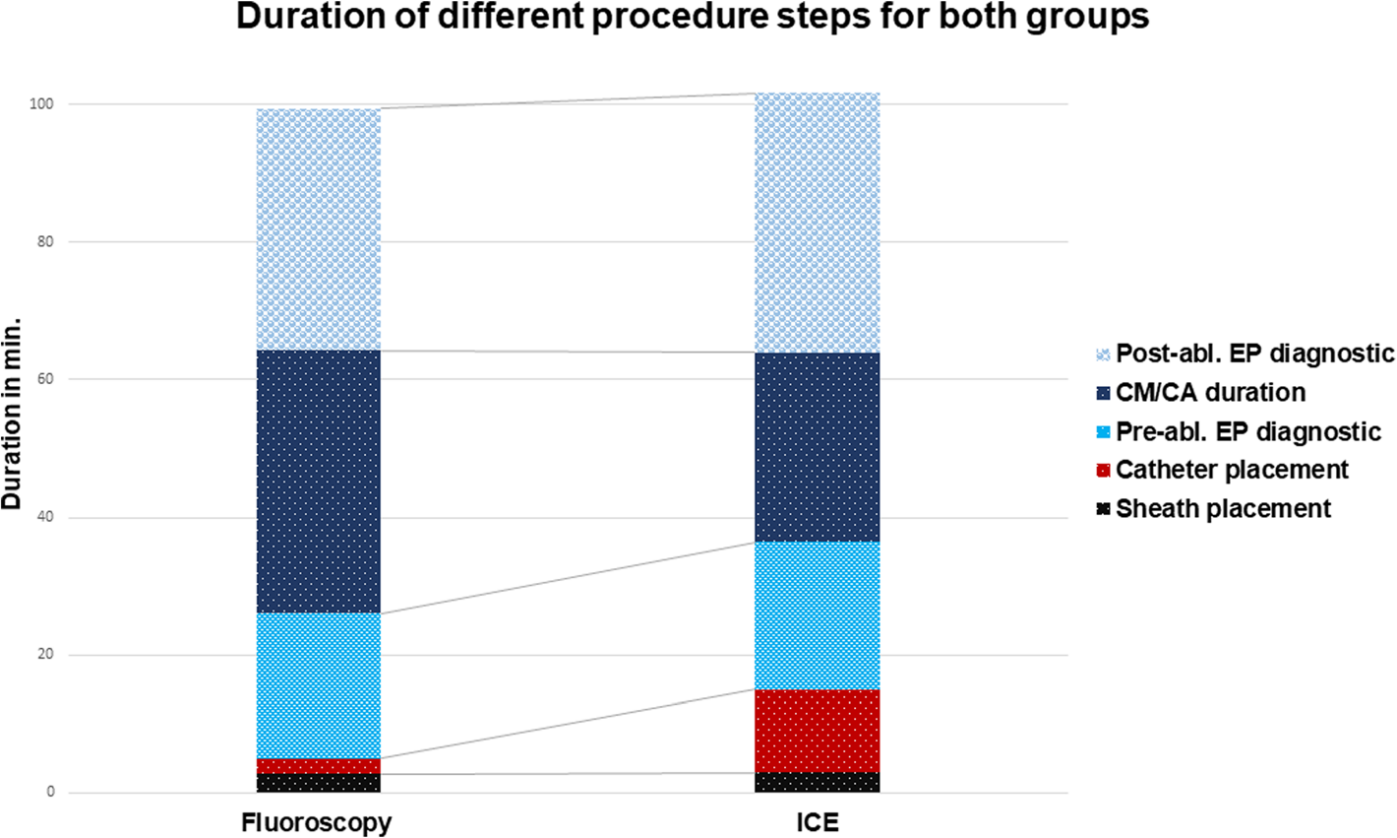
Duration of different procedure steps for both groups

Mean EPS duration in the zero-fluoroscopy group was 101.6 ± 40.2 min, whereas EPS duration in the fluoroscopy group was 99.4 ± 37.2 min (*p* = n.s.). Time needed for catheter placement was significantly longer in the zero-fluoroscopy group (12.0 ± 7.5 min vs. 2.2 ± 1.6 min, *p* <  0.05), though it became shorter with increasing operator experience. Mean catheter placement duration in the first half of the zero-fluoroscopy group (patients 1 to 11) was significantly longer as compared to the second half (patients 12 to 22) of the same group (14.3 ± 2.1 vs. 9.6 ± 2.6 min, *p* = 0.004). Figure 2 shows the detailed duration of different procedural steps for each patient in the zero-fluoroscopy group. On the other hand, cryo-application time (as defined above) was significantly shorter in the zero-fluoroscopy group (27.5 ± 37.0 min vs. 38.1 ± 33.9 min, *p* < 0.05). Figure 3 shows the duration of different procedural steps for both groups.

Mean CM and CA applications in the zero-fluoroscopy group were numerically lower as compared to those in the fluoroscopy group (CM: 7.5 ± 5.7 vs. 8.8 ± 6.2, *p* = n.s.; CA: 3.1 ± 1.7 vs. 3.2 ± 2.0, *p* = n.s.), after excluding cryo application attempts automatically aborted by the system, which occurred almost exclusively during CM.

During CM one temporary first degree AV block was observed in each group, both resolving within a few seconds after discontinuation of cryothermal delivery. In both patients CM and CA were successful at a more posterior location. Periprocedural vascular or cardiac complications were not observed in both groups. Continuous visualization of the blood flow direction by color doppler and adequate adjustment of the ICE-catheter tip to the vascular and cardiac structures allowed a safe navigation of the ICE-catheters with no vascular or cardiac complications, such as dissection, perforation or tamponade. Furthermore, using the steering lock for the ICE-catheter deflection helped to maintain a stable position of the catheter, avoiding catheter induced extrasystoles or other confounding arrhythmias.

After a mean follow up of 15.0 ± 4.2 months three (6.4%) patients [one (4.5%) in the zero-fluoroscopy and two (8.0%) in the fluoroscopy group] suffered an arrhythmia recurrence. All three patients reported recurrence of the clinical symptoms at the first scheduled outpatient control visit (after 3 months). Two of the three patients with arrhythmia recurrence showed an abolition of the slow pathway conduction after the last CA at the index procedure (= no AH jump or atrial echo), whereas the remaining patient showed a residual, but non-repetitive slow pathway conduction (= jump with max. One atrial echo). The two patients with arrhythmia recurrence in the fluoroscopy group underwent a second fluoroscopy guided procedure with successful CA of the slow pathway. The patient with arrhythmia recurrence in the zero-fluoroscopy group refused an immediate redo procedure due to less frequent arrhythmia episodes with improved health condition.

## Discussion

In this study we demonstrated comparable efficacy and safety of ICE mediated zero-fluoroscopy EP catheter navigation to traditional fluoroscopic EP catheter navigation for cryothermal slow pathway ablation in symptomatic AVNRT patients. Acute and long-term success, as well as access site or overall complication rates during cryothermal slow pathway ablation were not compromised by the implementation of a new zero-fluoroscopy navigation technique requiring an additional catheter for visualization of EP catheters and vascular or cardiac structures. Furthermore, we demonstrated that ICE mediated direct visualization of catheters and endocardial structures within the triangle of Koch shortens the cryo-application duration, though time needed for catheter placement is longer when compared to conventional fluoroscopic guidance, which results in similar mean EPS duration with both navigation techniques.

Catheter ablation under fluoroscopic control is the treatment of choice in patients with symptomatic supraventricular tachycardias [14]. In previous studies, higher malignancy rates for patients and EP lab staff were observed even with relatively low radiation doses during EP procedures [3, 4, 15]. Furthermore, the risk of skeletal disorders increases in operators and assistance staff wearing x-ray protection equipment [6]. This led electrophysiologists to a continuous struggle to avoid fluoroscopy and x-ray protection equipment during EP procedures. Several non-fluoroscopic catheter navigation techniques have emerged with 3D EAM being the most used one [7–10, 16–20]. The use of 3D EAM systems in ablation procedures of AVNRT patients was first reported in the late 90s [21, 22]. In a large meta-analysis comparing zero- or near-zero fluoroscopy with conventional fluoroscopy use during ablation of cardiac arrhythmias, Yang and colleagues reported similar acute or long-term success rates and procedure duration [23]. Accordingly, in our trial comparing a new zero-fluoroscopy imaging technique with the conventional fluoroscopy for cryothermal ablation of the slow pathway in AVNRT patients we confirm similar procedure duration with both techniques. Considering the different procedure steps, we observed a significantly shorter cryo-application duration under ICE guidance. This was mainly due to the direct visualization of the position of the ablation catheter within the triangle of Koch and its relationship to the fixed diagnostic catheters (in particular to the His recording catheter), which made targeting the slow pathway region considerably easier, especially if the anatomical approach (or a combination of the anatomical approach with the electrocardiographic one) was applied. Previously, Fisher and Batra independently reported on the advantage of ICE imaging while targeting the slow pathway region during radiofrequency ablation in AVNRT patients [24, 25]. In our trial, the number of CM attempts for localization of an effective and safe ablation region were numerically but not significantly lower in the ICE guided group. However, a shorter cryo-application duration in the zero-fluoroscopy group confirm the advantage of ICE visualization in targeting the anatomical region for effective slow pathway ablation. In contrast to cryo-application duration, the placement of EP and ICE catheters in the zero-fluoroscopy group was more difficult and took significantly longer, as compared to the same duration in the fluoroscopy group. While the septal aspect of the tricuspid ring including the ostium and proximal aspect of the coronary sinus could be easily visualised by an anterior angulation and a clockwise torque of the ICE probe, placing the diagnostic catheters (especially a decapolar diagnostic catheter into the coronary sinus) under solely ICE guidance was sometimes challenging, but catheter placement time seemed to decrease with operator’s experience. Similarly, in a recently published multicenter trial Pani and colleagues reported that operator experience is an important predicting factor in the achievement of zero-fluoroscopy catheter ablation of cardiac arrhythmias by other imaging technologies [7]. Engaging the steering lock of the ICE-catheter deflection after visualization of the target substrate helped keeping a stable position of the ICE catheter throughout the cryo-application, allowing a single-operator performance in most patients. Nevertheless, in patients with vascular tortuosity or special anatomy assistance staff helped to maintain the ICE-catheter in the required position, especially if the investigator had to use both hands to stabilize the ablation catheter before cryoadhesion had occurred.

The additional implementation of an 8 or 10F sheath (and the respective 2D- or 3D ICE catheter) in the zero-fluoroscopy group was not associated with periprocedural vascular or other complications, as they were not observed in both groups. On the contrary, real-time visualization of vascular and cardiac structures might have revealed earlier potential complications such as perforation, pericardial effusion or thrombus formation. Continuous visual control by ICE contributed even to a lower stress level for operators because of the real-time confirmation of cryoadhesion and consequently catheter stability during cryo applications (CM and CA), especially if the ablation substrate was located anteriorly in the triangle of Koch.

Patients with other or non-inducible supraventricular arrythmia were not included in the ICE&Ice trial. Zero-fluoroscopy EPS relying solely on ICE guidance might be achievable as well in patients with tachycardia mechanisms other than AVNRT, but in our pilot ICE&Ice trial (dealing with a new approach, in which literature, illustrations or experience with EP catheter navigation using ICE guidance were limited) we focused our investigation on patients with AVNRT. Larger investigator experience might increase the use of ICE guidance to achieve zero-fluoroscopy EPS, especially (but not only) in suspected arrhythmia cases with known ablation substrate, such as AVNRT, typical atrial flutter etc.

As expected, not only acute but also long-term results of cryothermal slow pathway ablation were similar in both groups, as we used identical protocols to define procedural success. Cryothermal ablation of the slow pathway has been associated with a comparable acute success but a higher recurrence rate when compared to radiofrequency ablation [1]. In a previous study, residual slow pathway conduction (patent AH- or VA-jump) at the end of a cryo-ablation procedure was associated with a higher recurrence risk as compared to abolition of slow pathway conduction [26]. However, two of the three patients with arrhythmia recurrence in our trial showed no slow pathway conduction at the end of the cryo-ablation procedure. Further investigation is warranted to identify specific risk factors for arrhythmia recurrence.

## Limitation

This is a retrospective data analysis of CA in AVNRT patients using two different catheter navigation techniques. The younger age in the ICE group is probably related to the investigator preference to offer a zero-fluoroscopy procedure more often to younger patients, which might have influenced the results of the study. Therefore, the represented data should be evaluated with caution because of the risk of related bias. Randomized prospective trials are warranted to investigate potential advantages of ICE-guided over fluoroscopy or 3D EAM mediated catheter navigation with respect to procedural performance and costs, acute or long term-success and complication rates.

## Conclusion

Zero-fluoroscopy ICE- guidance shows comparable efficacy and safety when compared to traditional fluoroscopic navigation during cryothermal ablation of the slow pathway in AVNRT patients. ICE visualization of catheters and endocardial structures within the triangle of Koch shortens the cryo-application duration, though time needed for catheter placement relying solely on ICE guidance is significantly longer, resulting in similar mean EPS duration with both navigation techniques.

## Additional files

Additional file 1: **Clip S1.** Advancement of the ICE-Catheter in the inferior caval vein. Colour doppler shows blood flow direction. Two other EP catheters are visualized within the vessel. (MOV 3648 kb)

Additional file 2: **Clip S2.** Cryoadhesion at posterior interatrial septum during Cryo-Ablation (6 o’clock) visualized by ICE. A diagnostic EP Catheter in anteroseptal position recording His-Potentials is visualized on the same echocardiographic plane (4 o’clock). (MOV 3609 kb)

## Data Availability

Due to ethical and legal restrictions with respect to patient privacy, investigators are not authorized to make the clinical data publicly available. The data will be available to interested researchers upon request by contacting Dr. Blerim Luani (bluani77@hotmail.com).

## Abbreviations

3D EAM: Three dimensional electroanatomical mapping
AVNRT: Atrioventricular-nodal-reentry-tachycardia
CA: Cryothermal ablation
CM: Cryo-mapping
DAVNP: Dual AV node physiology
ECG: Electrocardiography
EP: Electrophysiology
EPS: Electrophysiological study
ICE: Intracardiac echocardiography
